# Hypothyroid-Induced Rhabdomyolysis: A Case Report

**DOI:** 10.7759/cureus.66679

**Published:** 2024-08-12

**Authors:** Jennifer A Walker, Zachary Miles

**Affiliations:** 1 Emergency Medicine, Baylor Scott & White All Saints Medical Center, Fort Worth, USA; 2 Emergency Medicine, Burnett School of Medicine at Texas Christian University, Fort Worth, USA; 3 Emergency Medicine, Baylor University Medical Center, Dallas, USA

**Keywords:** non specific complaints, anchoring bias, acute kidney injury, hypothyroidism, non-traumatic rhabdomyolysis

## Abstract

Patients frequently present to the emergency department (ED) with non-specific complaints such as body aches and generalized weakness, which can have an extensive differential diagnosis. Hypothyroidism and rhabdomyolysis are known causes of generalized weakness and body aches but are usually considered separate entities.

In this article, we describe a patient who presented to the ED with symptoms including generalized weakness and muscle aches and was diagnosed with rhabdomyolysis. She presented days later with ongoing, worsening symptoms and was diagnosed with hypothyroid-induced rhabdomyolysis and acute kidney injury.

Patients who present with non-specific complaints may have delayed diagnoses that can lead to progression of their disease. Patients with hypothyroidism can develop non-traumatic rhabdomyolysis which can later lead to acute kidney injury. This case illustrates the importance of keeping a wide differential when evaluating patients with generalized complaints and recognizing hypothyroidism as a potential cause of rhabdomyolysis.

## Introduction

Patients present frequently to the emergency department (ED) with non-specific complaints. Nemec et al., for example, found in their 2010 study that 13.5% of non-trauma patients present with vague complaints such as generalized weakness or feeling unwell [1]. That same study found in a 30-day follow-up, that 59% of those patients were later diagnosed with a serious condition, and their mortality rate was as high as 6% [1]. Nickel et al., illustrated that patients present to the ED with generalized weakness as a noted complaint in nearly 20% of visits [2]. Such non-specific symptoms, while difficult to work up, have high rates of morbidity and increased length of hospital stay [1,3,4]. It is imperative to have a high index of suspicion for serious illness, even when the presenting complaints are vague, and to avoid anchoring bias when these patients return to the ED with ongoing symptoms.

Generalized weakness and myalgias are non-specific complaints that have several serious causes that should be considered, including cardiovascular etiologies, electrolyte abnormalities, infection, anemia, rhabdomyolysis, hypothyroidism, and adrenal insufficiency [5-7]. 

Hypothyroidism as a specific cause of weakness and myalgias, often has a long and insidious onset, with the primary symptoms caused by decreased metabolic rate and diminished sympathetic excitability. Chills, fatigue, swelling, lethargy, and menstrual disorders are common complaints [6]. Rhabdomyolysis, on the other hand, frequently presents with the triad of myalgia, muscle weakness, and dark urine, with muscle pain being the most common symptom [8]. Rhabdomyolysis is generally associated with risk factors such as heavy exercise, inflammatory processes, toxins, or certain medications [9,10]. Hypothyroidism-associated rhabdomyolysis, however, is less frequently discussed in the literature [11-22]. 

In this case report, we present a patient with non-traumatic rhabdomyolysis associated with hypothyroidism and discuss several other cases in the literature as a reminder of this specific pathophysiologic association and as an important example of maintaining a broad differential diagnosis and avoiding anchoring bias when evaluating non-specific-complaints.

## Case presentation

A woman in her 30s with comorbidities including seizures and anxiety presented to the ED with complaints of diffuse muscle cramping and weakness for several months. Five months prior, she was diagnosed with thyrotoxicosis related to Grave’s disease and was subsequently treated with radioablation therapy. One month later, she began to experience intense muscle pain and spasms. She also experienced bilateral lower extremity edema. She reported that she followed up with her endocrinologist but was told everything was within normal limits. She presented to the ED once and was given IV fluids and analgesia; however, symptoms persisted. She returned to the ED a second time, and laboratory workup is noted in Table 1, with notable, mild elevation in creatine kinase (CK) levels.

**Table 1:** Laboratory values at emergency department visit where initial rhabdomyolysis was diagnosed WBC: white blood cells, WNL: within normal limits, RBC: red blood cells, BUN: blood urea nitrogen, HCG: human chorionic gonadotropin.

Laboratory Test	Laboratory Value	Reference Values
WBC	9.8 k/uL (differential WNL)	4.5-11.0K/uL
Hemoglobin	13.3 g/dL	13.5-18.0g/dL
Platelet Count	362 k/uL	140 - 440 K/uL
Urine Glucose	Negative	Negative
Urine Ketones	Negative	Negative
Urine Blood	Large	Negative
Urine Nitrite	Negative	Negative
Urine Bilirubin	Negative	Negative
Urobilinogen	0.2	Negative
Urine Leukocyte Esterase	Negative	Negative
Urine RBC	11-50	Negative
Urine WBC	1-3	Negative
Urine Epithelial Cells	Many (10-100)	Negative
Urine Bacteria	Few (1-3)	Negative
Sodium	138.5 mmol/L	136 - 145 mmol/L
Potassium	3.6mmol/L	3.5 - 5.1 mmol/L
Chloride	103	98 - 107 mmol/L
Carbon Dioxide	27.6 mmol/L	22 - 29 mmol/L
Anion Gap	11	
BUN	9 mg/dL	7-18 mg/dL
Creatinine	1.1 mg/dL	0.6-1.2 mg/dL
Glucose	88mg/dL	70-110 mg/dL
Calcium	8.3 mg/dL	8.4-10.2 mg/dL
Phosphorus	3.6mg/dL	3.0-4.5 mg/dL
Magnesium	1.9 mg/dL	1.5-2.0 mEq/L
Total Creatine Kinase	2747 U/L	10-70 U/L
Urine HCG	Negative	

She was diagnosed with mild rhabdomyolysis and was given intravenous (IV) ketorolac and later reported that her muscle pain had improved. She was discharged home and treated as an outpatient with acetaminophen with codeine. 

She continued to have severe myalgias and had “Coca-Cola” colored urine, so she presented to another ED where she advised providers about her recent diagnosis of rhabdomyolysis. Documentation from that visit is not available, but per the patient, the provider noted hematuria and diagnosed her with a urinary tract infection.

Seven days after the initial diagnosis of rhabdomyolysis, she presented to the ED with worsening symptoms, not improved by outpatient treatment. She was having such severe pain and weakness, that she collapsed and was unable to ambulate and was brought in by a concerned acquaintance. Her review of systems (ROS) was positive for fatigue, light-headedness, paresthesias, generalized weakness, and lower extremity edema. She also endorsed abdominal pain and dark urine. She stated that she felt exhausted and that it hurt to move, and she specifically stated that she was afraid to lift her arms above her shoulders because it would “trigger the pain” which was described as fasciculation-type movements. She did not report significant new or vigorous exercise or other muscle injury. She stated that she was frustrated and anxious that providers were not believing her symptoms.

During that visit, her vital signs were documented as a temperature of 36.7 °C (98.1 °F), heart rate (HR) 55-74, blood pressure (BP) 158/116, respiratory rate (RR) 20, and saturation of peripheral oxygen (SpO2) 100% on room air. Physical examination documented that she appeared ill, sleepy but anxious. She had normal cardiac, pulmonary, and abdominal exams. Her neurological exam was also documented as grossly normal, including extremity strength, except that she was unable to lift her arms due to reports of pain. Pertinent laboratory results from that visit are demonstrated in Table 2.

**Table 2:** Laboratory values at repeat emergency department visit SGOT: serum glutamic-oxaloacetic transaminase, AST: aspartate aminotransferase, RBC: red blood cells, TSH: thyroid stimulating hormone, WBC: white blood cells.

Laboratory Test	Laboratory Value	Reference Values
Creatinine	1.49 mg/dL	0.6-1.2 mg/dL
Alkaline Phosphatase	183 U/L	25-100 U/L
SGOT (AST)	51 U/L	12-38 U/L
Total Creatine Kinase	2069 U/L	10-70 U/L
Urine Protein	Positive 3+	Negative
Urine Gross Blood	Positive 3+	Negative
Urine RBC	16-30	Negative
TSH	81.70	0.4-4.0 uU/mL
Free T4	<0.15	0.9-1.7 ng/dL
WBC	11.4 K/uL	4.5-11.0K/uL
Hemoglobin	14.0 g/dL	13.5-18.0g/dL
Platelet Count	369 K/uL	140 - 440 K/uL

She was admitted to the medicine service with the diagnosis of severe hypothyroidism complicated by hypothyroid myopathy with ongoing rhabdomyolysis and acute kidney injury. Further workup of her acute kidney injury, proteinuria, and microscopic hematuria included a renal biopsy notable for IgA nephropathy and 30% glomerulosclerosis. During admission, she received one liter of IV crystaloid bolus, followed by maintenance fluids at 125 cc/hour. She was initiated on levothyroxine 25 mcg PO daily for three days, followed by an increase to 50 mcg PO. She also received solumedrol 1 gram IV for three days, then was started on oral prednisone 60 mg daily with plans to decrease the dose by 0.2mg/kg monthly for four months. At the time of discharge from the hospital, her renal function, creatine kinase (CK) levels, and myalgias had improved. 

## Discussion

The prevalence of hypothyroidism in the United States is approximately 4.6%, of which 4.3% are considered subclinical cases. About 0.3% of cases are described as overt hypothyroidism [23]. Meanwhile, the true incidence of rhabdomyolysis is largely unknown due to its variable clinical presentation and under-recognition. Only about 26,000 cases are reported each year [24]. Hypothyroidism associated rhabdomyolysis without other risk factors such as medication use, heavy exercise, or trauma, is a rare condition with only a limited number of documented cases, and a review of the literature is summarized in Table 3 [8,11-17,19,20]. The majority of these patients were male (70%) with an age range of 24-44 years old at diagnosis. Presenting CK levels ranged from 1,364 to 21,644, and while these studies described thyroid tests with inconsistent reference ranges, the majority of these cases revealed Free T4 levels that were below their labs' limits (60%). These cases overwhelmingly described myalgias, weight gain, edema, and weakness as presenting symptoms of hypothyroidism-related rhabdomyolysis.

**Table 3:** List and description of other case reports involving hypothyroid-related rhabdomyolysis

	Age	Gender	PMH	Symptoms	Diagnosis
Janjua et al. [8]	36	Male	None	Myalgias, alopecia, weight gain	AKI due to hypothyroidism-related rhabdomyolysis
Munankami et al. [11]	24	Male	None	Myalgias, weakness, weight gain, constipation	Hypothyroid-related rhabdomyolysis from Hashimoto’s thyroiditis
Syed et al.[12]	34	Female	Hypothyroid	Fatigue, muscle cramps	Rhabdomyolysis from untreated hypothyroidism
Baghi et al. [13]	42	Male	None	Myalgias, dry skin, facial edema, globus sensation	AKI due to hypothyroid-related rhabdomyolysis from Hashimoto’s thyroiditis
Mohamed et al. [14]	44	Male	None	Delusions, hallucinations, constipation, myalgias	Rhabdomyolysis and Hashimoto’s encephalopathy
Gurala et al. [15]	35	Male	None	Myalgias, fatigue, weight gain	Rhabdomyolysis from undiagnosed hypothyroidism
Neves et al. [16]	40	Female	HTN, Hypothyroid	Myalgias, weakness, weight gain, edema, dry skin	AKI due to hypothyroidism-related rhabdomyolysis
Nikolaidou et al. [17]	41	Female	None	Edema, insomnia, alopecia, weight gain, constipation, amenorrhea, hoarse voice	AKI due to hypothyroidism-related rhabdomyolysis
Kisakol et al. [19]	19	Male	None	Myalgias, edema, weakness	Hypothyroid-related rhabdomyolysis
Bhansali et al. [20]	41	Male	HTN, Autoimmune thyroiditis	Edema, myalgias, weakness, oliguria	Rhabdomyolysis from untreated hypothyroidism

The mechanism connecting hypothyroidism with muscle tissue breakdown, and thus rhabdomyolysis, is not well understood. One suggested theory is that low T3 production decreases glycogen utilization, causing adenosine triphosphate (ATP) hydrolysis and reduced mitochondrial activity, leading to a lack of substrate required for muscle activation and further metabolic dysfunction [25-27]. Additional mechanisms including the transition from fast-twitch (type II) muscle fibers into slow-twitch (type I) muscle fibers have also been explored [19,28]. Thyroid hormone is thought to have a role in modulating miR-133a1, a gene responsible for regulating this muscle type conversion [29]. 

Since the incidence of hypothyroid-associated rhabdomyolysis is low, it is difficult to confirm other risk factors. Statin therapy, advancing age, diabetes mellitus, liver disease, renal impairment, and alcoholism are posited as potential risk factors for hypothyroid-induced rhabdomyolysis [13,21,22] since they are already risk factors for rhabdomyolysis, but all of the cases we included did not have these comorbidities.

In terms of the severity of rhabdomyolysis, Ghayur described that no specific severity of hypothyroidism is associated with the severity of rhabdomyolysis [30]. This appears so in our included references. For example, Munankami et al. demonstrated presenting CK levels of 1,364 U/L with less than the reference range of Free T4 (<0.25 ng/dl), but Bhagi et al. described presenting CK levels of 21,644 U/L with less than the reference range of Free T4 (<0.5 ng/dl) [11,13]. Notably, the American Thyroid Association (ATA) does recommend that patients with elevated CK levels - particularly if present for longer than two weeks, if previously normal during a euthyroid state, or if risk factors for thyroid disease are present - should have thyroid studies evaluated [31]. 

Rhabdomyolysis is known to lead to acute kidney injury through multiple mechanisms [32]. Myoglobin released during rhabdomyolysis causes renal vasoconstriction. There are direct cytotoxic effects of myoglobin in the renal tubules, and plaque formation in the renal tubules can cause blockage. Interestingly, our patient had a further workup for her acute kidney injury which included a biopsy noting IgA nephropathy, and we did find one other case report demonstrating a young woman with hypothyroidism and elevation in CK levels in combination with IgA nephropathy [33]. 

## Conclusions

Hypothyroid-associated rhabdomyolysis can be insidious and difficult to diagnose. The patient in our case presented to multiple medical providers with non-specific complaints of myalgias and generalized weakness. She did not have well-known risk factors for rhabdomyolysis such as heavy exercise, trauma, or significant medication use, and she had several points of contact with the medical system without a complete diagnosis.

Initially, she was diagnosed with non-traumatic rhabdomyolysis, and her symptoms improved after hydration and pain medications. She returned seven days later, however, with worsening symptoms and workup demonstrating ongoing rhabdomyolysis but also severe hypothyroidism and acute kidney injury. We believe this is one of only several other documented cases related to hypothyroid-associated rhabdomyolysis. There are proposed mechanisms that fit into a pathophysiologic model, and the ATA does recommend checking thyroid studies in patients with persistent CK elevation. 

This case and the other referenced cases overwhelmingly reflect myalgias, weight gain, edema, and weakness as presenting symptoms of hypothyroidism-related rhabdomyolysis, and it is important to consider this diagnosis when patients present with this combination of symptoms, especially if non-traumatic rhabdomyolysis is diagnosed. This case highlights the importance of avoiding anchoring bias when re-evaluating patients for ongoing complaints and keeping a broad differential diagnosis when assessing non-specific complaints. It also highlights the importance of being aware of the uncommon, but documented association of rhabdomyolysis and hypothyroidism. 
